# Preparation of Cobalt–Nitrogen Co-Doped Carbon Nanotubes for Activated Peroxymonosulfate Degradation of Carbamazepine

**DOI:** 10.3390/molecules29071525

**Published:** 2024-03-28

**Authors:** Bei Chu, Yixin Tan, Yichen Lou, Jiawei Lin, Yiman Liu, Jiaying Feng, Hui Chen

**Affiliations:** Ningbo Key Laboratory of Agricultural Germplasm Resources Mining and Environmental Regulation, College of Science and Technology, Ningbo University, Cixi 315300, China; tanyixin789@126.com (Y.T.); lyc224840123@126.com (Y.L.); aw444666777888@163.com (J.L.); liuyiman_2024@163.com (Y.L.); yingjia0327@163.com (J.F.); chenhui07@126.com (H.C.)

**Keywords:** carbamazepine, peroxymonosulfate, advanced oxidation process, carbon nanotubes

## Abstract

Cobalt–nitrogen co-doped carbon nanotubes (Co3@NCNT-800) were synthesized via a facile and economical approach to investigate the efficient degradation of organic pollutants in aqueous environments. This material demonstrated high catalytic efficiency in the degradation of carbamazepine (CBZ) in the presence of peroxymonosulfate (PMS). The experimental data revealed that at a neutral pH of 7 and an initial CBZ concentration of 20 mg/L, the application of Co3@NCNT-800 at 0.2 g/L facilitated a degradation rate of 64.7% within 60 min. Mechanistic investigations indicated that the presence of pyridinic nitrogen and cobalt species enhanced the generation of reactive oxygen species. Radical scavenging assays and electron spin resonance spectroscopy confirmed that radical and nonradical pathways contributed to CBZ degradation, with the nonradical mechanism being predominant. This research presents the development of a novel PMS catalyst, synthesized through an efficient and stable method, which provides a cost-effective solution for the remediation of organic contaminants in water.

## 1. Introduction

Pharmaceuticals and personal care products are increasingly recognized as emergent contaminants within aquatic environments, posing potential risks to wildlife and human health [1,2]. Traditional treatment methodologies, including physical [3,4], chemical [5], and biological approaches [6], are often inadequate for the removal of these pollutants, particularly at low concentrations [7,8]. Carbamazepine (CBZ), a widely used anticonvulsant and mood-stabilizing drug with an annual global consumption of approximately 1214 tons, is a persistent organic pollutant [9]. As CBZ is not fully absorbed by the human body, it is excreted and enters the environment as various metabolites [10]. Its recalcitrant nature leads to its pervasive presence in diverse water bodies [11]. Notably, chronic environmental exposure to CBZ has been associated with severe human health implications, including coma and fatalities [12]. Furthermore, CBZ adversely affects aquatic life, vegetation, and microbial communities, thereby disrupting the equilibrium and integrity of aquatic ecosystems [13]. Consequently, the development of innovative and effective remediation technologies for emerging pharmaceutical contaminants, particularly for the advanced treatment of CBZ, is necessary for safeguarding ecological systems and public health [14,15].

Advanced oxidation processes (AOPs) represent a potent suite of technologies for the oxidative degradation of pollutants via reactive oxidative species, such as hydroxyl (•OH) and sulfate radicals (SO_4_•^−^) [16,17,18]. Traditional AOPs predominantly generate hydroxyl radicals as oxidants to decompose organic contaminants, a subset known as hydroxyl-radical-based AOPs (HR AOPs) [19,20]. The efficacy of HR AOPs is influenced by pH, leading to variable oxidation performance [21]. By contrast, sulfate-radical-based AOPs (SR AOPs) demonstrate robust performance across a range of complex aqueous environments [22]. SR AOPs require activators such as peroxydisulfate (PS) and peroxymonosulfate (PMS) [23]. In particular, PMS features a more asymmetric molecular structure and elongated O–O bonds than peroxydisulfate, which results in a lower energy requirement for bond cleavage and free radical generation [24]. This characteristic renders it more effective for the treatment of wastewater containing persistent pharmaceuticals such as CBZ [25].

Carbon-based materials have emerged as superior alternatives to traditional metal-based catalysts because of their expansive specific surface area, intricate porous architecture, abundant functional groups, and environmental compatibility [26]. Among these, certain carbon nanomaterials such as reduced graphene oxide, activated carbon (AC), and carbon nanotubes (CNTs) have demonstrated efficacy in catalyzing the degradation of aqueous pollutants via persulfate (PS) activation [27,28]. Carbon nanotubes, which are characterized by their one-dimensional, tubular structure composed of single or multiple layers of graphene, represent a novel class of carbon materials [29]. With diameters in the nanometer range and lengths varying from tens of nanometers to several millimeters, CNTs boast a unique set of properties, including a distinctive hollow structure, adsorption capacity, and electrical conductivity. However, the intrinsic catalytic performance of pristine CNTs is moderate, and it lacks stability [30]. Thus, researchers have turned their attention to the multiscale engineering of carbon nanostructures to enhance the catalytic efficiency of CNTs, focusing on sp2 hybridization patterns, structural design, and chemical composition alterations. Doping with heteroatoms such as nitrogen (N), boron (B), and sulfur (S) has been particularly effective, often resulting in a significant increase in catalytic activity that can exceed that of popular metal-based catalysts [31,32]. This improvement is attributed to the ability of heteroatom doping to modulate the electronic characteristics of the native sp2-bonded carbon, enlarge the specific surface area, and create novel active sites [33]. Nitrogen doping is the most extensively investigated form of heteroatom incorporation [34]. It alters the electron density around the carbon atoms within the CNTs, thereby endowing them with exceptional conductivity [35]. Moreover, the electronic effects induced by nitrogen doping contribute to a distinctive catalytic performance, which is increasingly leveraged in environmental applications [36].

At present, the synthesis and functionalization of CNTs require an intricate apparatus, costly precursors, and elaborate procedures [37]. The in situ generation of CNTs using biochar substrates presents an economical, eco-friendly, and time-efficient alternative. For the in situ fabrication of nitrogen-doped CNTs, nitrogen atoms are integrated into the carbon matrix, facilitating the formation of nanotubes [38]. In general, transition metals serve as catalysts in the synthesis of nitrogen-rich organic precursors for the in situ growth of nitrogen-doped CNTs, with cobalt being a particularly effective agent. Recent studies have indicated that cobalt-based catalysts exhibit superior activation effects on PMS compared with their iron-based counterparts [39]. Nonetheless, there is a paucity of literature on the in situ synthesis of cobalt–nitrogen (Co–N) co-doped nanotubes specifically as activators for PS and PMS.

In this investigation, a cost-effective and straightforward approach was used to synthesize a Co–N-doped CNT catalyst using AC derived from soybean dregs for the degradation of CBZ in aqueous solutions. The physicochemical properties of the catalyst, including crystal structure, micromorphology, surface functional groups, specific surface area, and elemental composition, were meticulously characterized through a series of analytical techniques such as X-ray diffraction (XRD), scanning electron microscopy (SEM), transmission electron microscopy (TEM), Brunauer–Emmett–Teller (BET) surface analysis, and X-ray photoelectron spectroscopy (XPS). This study further examined the influence of various parameters on the catalytic performance of the C–N co-doped CNTs, including catalyst and PMS dosages, initial solution concentration, pH, the presence of coexisting anions, and humic acid (HA). The active species involved in the degradation process were probed using radical scavengers, and electron paramagnetic resonance (EPR) was used to identify the radicals generated within the system, thereby elucidating the degradation mechanism. In addition, liquid chromatography mass spectrometry (LC–MS) was utilized to investigate the intermediate degradation products of CBZ in the Co3@NCNT/PMS system, providing insights into the catalytic breakdown pathway.

## 2. Results and Discussion

### 2.1. Characterization of the Catalysts

**Figure 1:**
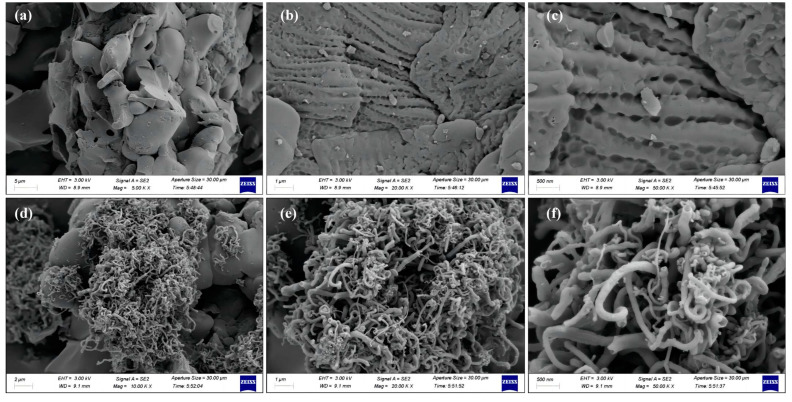
SEM images of sample BZ (**a**–**c**) and Co3@NCNT-800 (**d**–**f**).

As shown in Figure 1, the microstructure and morphology of the synthesized AC samples were examined by SEM. The AC labeled as BZ exhibits a disordered, flaky structure characteristic of AC, with a notably loose and porous internal composition. Concurrently, the Co3@NCNT-800 sample revealed a profusion of CNTs adorning the surface of the AC, indicating that the synthesis successfully generated CNTs and facilitated their proliferation on the AC substrate.

**Figure 2:**
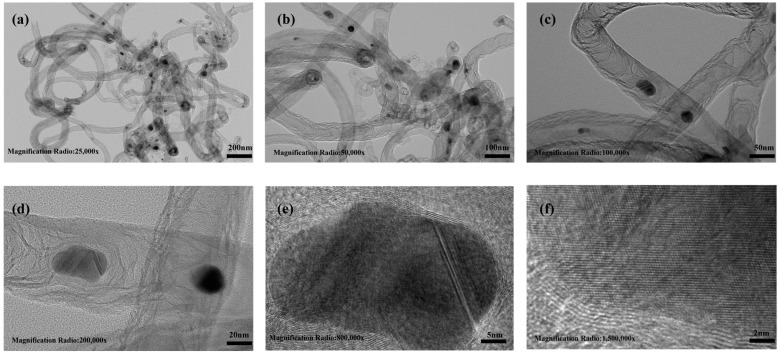
TEM images of Co3@NCNT-800 (Magnification ratio: 25,000× (**a**); 50,000× (**b**);100,000× (**c**); 200,000× (**d**); 800,000× (**e**); 1,500,000× (**f**)).

In further elucidating the morphology and structure of the catalyst, TEM was performed, and the findings are presented in Figure 2. As shown in Figure 2a–f, Co3@NCNT-800 has a tubular configuration reminiscent of bamboo, complete with sequential “bamboo knots”, each exhibiting distinct morphologies. The interiors of these tubes are lined with a granular material, and the tube walls are composed of multiple layers. The results indicate that the tubes have nanometric diameters and lengths extending to several microns. The nanotubes are demarcated by bamboo knots, forming “small compartments” of varying lengths, with individual compartments measuring between 20 and 50 nm. The external wall thickness of the tubes is approximately 2 nm, aligning with the structure of multiwalled CNTs akin to bamboo. As shown in Figure 2a, particles with an average diameter of approximately 20 nm are affixed to the exterior of the tube walls. Closer examination of the high-resolution images shown in Figure 2e,f reveals discernible lattice fringes. The lattice fringes could be attributed to the (002) planes of graphitic carbon [40].

**Figure 3:**
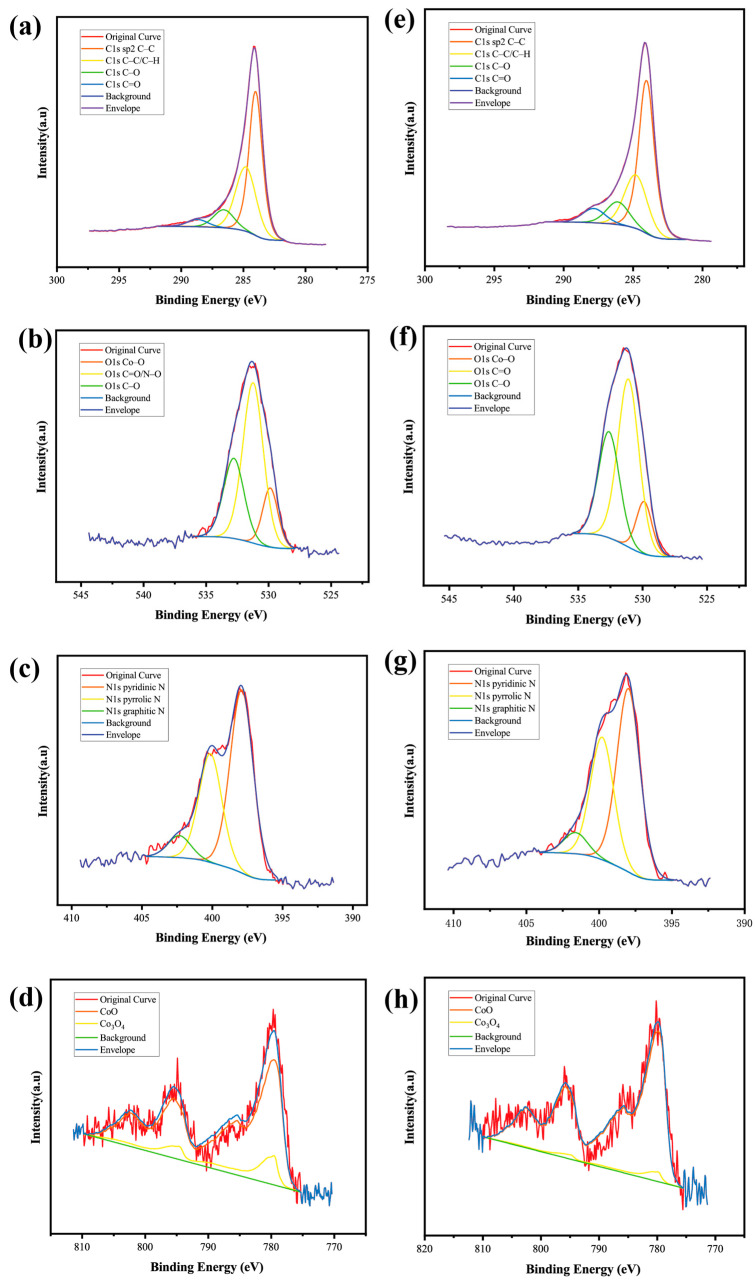
The C1s, O1s, N1s, and Co2p XPS spectra of Co3@NCNT-800 (before reaction) (**a**–**d**); and the C1s, O1s, N1s, and Co2p XPS spectra of Co3@NCNT-800 (after reaction) (**e**–**h**).

In this study, the composition and catalytic mechanism of the Co3@NCNT-800 catalyst were elucidated. The XPS orbital spectra of Co3@NCNT-800 were thoroughly analyzed. The comprehensive scanning spectrum (Figure S1) illustrates the catalytic state before and after the reaction. Characteristic binding energy peaks were observed at 284.39 eV for C1s, 398.73 eV for N1s, 531.46 eV for O1s, and 780.02 eV for Co2p [41]. Table S1 presents a comparative analysis of the surface elemental composition and content of Co3@NCNT-800 before and after the catalytic reaction. In addition, Figure 3a–h displays the changes in the composition of the N-doped CNT coupled with cobalt (Co3@NCNT-800) because of the reaction. For the C1s orbital region, Figure 3a identifies the presence of sp2-hybridized C–C, C–C/C–H, C–O, and C=O bonds at binding energies of 284.00, 284.80, 286.57, and 288.67 eV, respectively [42]. The detection of C–O bonds indicates the successful incorporation of oxygen into the CNT framework, as supported by previous studies. The O1s orbital region (Figure 3b,f) reveals the presence of Co–O, C=O, and C–O bonds at 529.87, 531.24, and 532.78 eV, respectively. The presence of these oxygen species is attributed to the oxidation of oxygen-containing functional groups on cobalt and CNTs. Post-reaction analysis indicates a decrease in the content of Co–O and C–O bonds, alongside an increase in C=O bonds. During the reaction, some C–O bonds may be transformed into C=O bonds, highlighting the dynamic changes in the surface chemistry of the catalyst during the catalytic process. Figure 3c,g presents the N1s orbital spectra, where distinct peaks corresponding to different nitrogen configurations are observed. In particular, pyridine N, pyrrolic N, and graphitic N were identified at binding energies of 397.98, 399.79, and 401.62 eV, respectively [43]. These nitrogen configurations, particularly pyridinic, pyrrolic, and graphitic nitrogen, may serve as active sites for PMS activation, as indicated by the literature, and could enhance the performance of the catalyst [40]. Table S2 details the quantitative changes in nitrogen configurations, noting a decrease in pyridine nitrogen content from 58.26% to 55.88% after the reaction. This reduction in pyridine nitrogen sites could lead to a diminished number of coordination sites available on the catalyst, which may attenuate its catalytic activity.

The Co2p orbital spectra (Figure 3d,h) reveal the valence state alterations of cobalt before and after the reaction. The spectra were deconvoluted using the nonlinear least squares fitting method. The fitting results indicate the predominance of cobalt oxide species, particularly CoO and Co_3_O_4_, within the spectra. The relative proportions of these species are listed in Table S2. A notable increase in the proportion of CoO and a concurrent decrease in Co_3_O_4_ postreaction indicate that a partial conversion of Co_3_O_4_ to CoO occurs during the catalytic process. This transformation indicates the changes in the oxidation state of cobalt, which may have implications for the catalytic activity and its mechanism of action.

The catalyst underwent BET analysis to determine its specific surface area and pore-size distribution, which were further characterized using nitrogen adsorption–desorption isotherms. The results of these analyses are presented in Figure S2 and Table 1. The BET surface area, pore size, and pore volume of the catalyst were calculated on the basis of the Barrett–Joyner–Halenda model. The findings indicate that the samples Co0@NCNT-800, Co1@NCNT-800, and Co3@NCNT-800 exhibit type-I isotherms, which are characteristics of a microporous structure. Among these, the Co1@NCNT-800 sample has the largest specific surface area of 715 m^2^/g. This substantial surface area is attributed to the catalytic growth of additional CNTs, which enhances the surface area. Conversely, the specific surface area for Co3@NCNT-800 is reduced to 614 m^2^/g. This decrease may be ascribed to pore blockage resulting from further cobalt doping.

**Figure 4:**
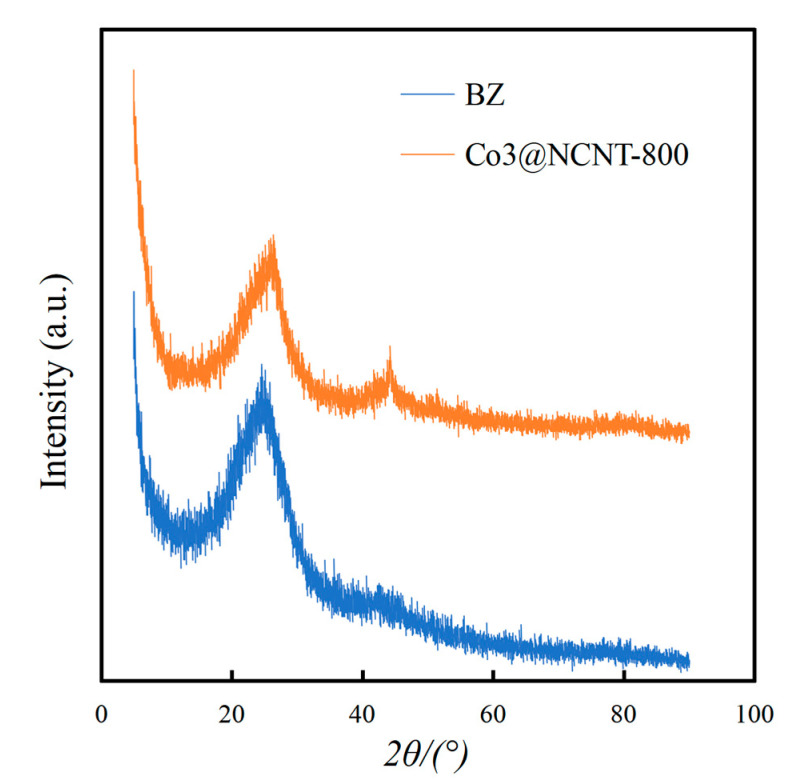
XRD spectra of BZ and Co3@NCNT-800.

Figure 4 presents the XRD patterns of BZ and Co3@NCNT-800 samples. All specimens exhibit a broad diffraction peak at approximately 23.6°, which is attributed to the (002) lattice plane of graphitic carbon. Notably, the Co3@NCNT-800 sample displays an additional broad peak at 44.4°, corresponding to the (100) plane of crystalline carbon. This feature indicates that Co3@NCNT-800 has a high level of graphitization [44].

### 2.2. Catalytic Performance Evaluation

**Figure 5:**
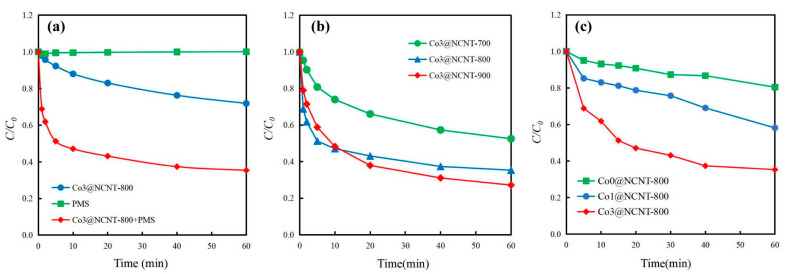
Degradation of CBZ by different catalysts (**a**); the degradation of CBZ by the Co3@NCNT−700/PMS, Co3@NCNT−800/PMS, and Co3@NCNT−900/PMS system (**b**); the degradation of CBZ by the Co0@NCNT−800/PMS, Co1@NCNT−800/PMS, and Co3@NCNT−800/PMS system (**c**).

Figure 5a illustrates the comparative efficacy of different catalysts in the degradation of CBZ. PMS alone exhibits limited capability in degrading CBZ, which is attributed to its relatively low oxidation–reduction potential and inability to self-activate, as reported in the literature [45]. Conversely, Co3@NCNT-800, when used independently, demonstrated a modest proficiency in CBZ removal, achieving approximately 29.0% degradation within 60 min. This level of degradation is approximately two times that of PMS alone, which could be attributed to the adsorptive properties of the Co3@NCNT-800 surface adsorption.

Remarkably, the presence of the Co3@NCNT-800/PMS system results in a substantial enhancement in the CBZ removal efficiency, with approximately 65% degradation achieved within the same timeframe. This significant increase indicates that Co3@NCNT-800 exerts a pronounced catalytic effect on PMS, facilitating the degradation of CBZ. The influence of the calcination temperature on the catalytic performance is depicted in Figure 5b. At a calcination temperature of 700 °C, Co3@NCNT-700 exhibits a suboptimal catalytic impact on PMS, with a CBZ removal rate of only 48%. An increase in the calcination temperature to 800 °C markedly improves the catalytic effect, with the removal rate of CBZ reaching 65%. Further increasing the temperature to 900 °C results in a continued, albeit marginal, enhancement of the catalytic performance, achieving a CBZ removal rate of 73%. This trend indicates that a calcination temperature of 800 °C effectively prevents the agglomeration and structural collapse of the CNTs, thereby promoting CBZ degradation. However, the incremental benefit diminishes with a further temperature increase, indicating an optimal calcination threshold for maximizing catalytic efficiency. Concurrently, the effect of varying the cobalt doping levels within the catalyst was investigated (Figure 5c). In the absence of cobalt doping, the catalyst designated as Co0@NCNT-800 exhibited a minimal catalytic influence on PMS, with a CBZ removal efficiency of merely 19%. By contrast, the Co1@NCNT-800 and Co3@NCNT-800 catalysts demonstrated enhanced catalytic activity toward PMS. In particular, the Co1@NCNT-800/PMS and Co3@NCNT-800/PMS systems achieved CBZ removal rates of 42% and 65%, respectively. These results indicate a positive correlation between the degree of cobalt doping and catalytic effectiveness. In summary, the Co3@NCNT-800/PMS system markedly enhances the degradation efficiency of CBZ. As shown in Table 2, Co3@NCNT-800 is significantly superior to the other catalysts presented in the references, making it an excellent candidate for exploring the impact of various environmental factors on catalytic processes.

### 2.3. Parameters Impacting CBZ Degradation by the Co3@NCNT-800/PMS System

**Figure 6:**
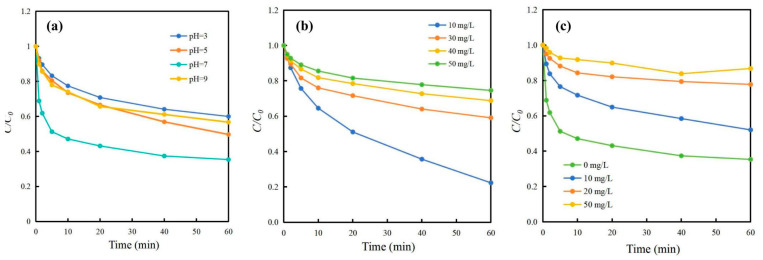
Influence of pH (**a**); CBZ concentration (**b**); and humic acid concentration (**c**).

The initial pH values of the four solutions were adjusted to 3, 5, 7, and 9 to determine the influence of the initial pH on the degradation of CBZ within the system. Figure 6a illustrates the impact of varying the initial pH levels on the degradation of CBZ in the Co3@NCNT-800/PMS system. As indicated by the data, the CBZ removal efficiencies were found to be 40.9% at pH 3, 50.4% at pH 5, 64.7% at pH 7, and 43.4% at pH 9. Therefore, high and low pH levels are detrimental to CBZ removal. This finding is attributed to the scavenging of reactive species by H^+^ ions at lower pH values and the reduced formation of reactive species at elevated pH levels [52].

Figure 6b demonstrates that an increase in CBZ concentration correlates with a decrease in the system’s CBZ degradation efficiency, as well as a deceleration in the degradation rate. This phenomenon is due to the constant quantity of a catalyst and PMS within the system, which implies that the generation of reactive species by the catalyst-activated PMS remains unchanged. Consequently, as the concentration of CBZ increases, there is heightened competition for oxidants between CBZ and its intermediate degradation products, leading to a reduced degradation rate of CBZ.

Natural organic matter (NOM), omnipresent in aquatic environments, is known to quench free radicals and other oxidative species, thereby diminishing the oxidative efficacy of treatment processes. In this investigation, HA was selected as a representative NOM to examine its impact on the degradation of CBZ. The experimental design included four distinct HA concentration levels: 0, 10, 20, and 50 mg/L. As depicted in Figure 6c, the introduction of HA impeded the degradation of CBZ. In particular, when the HA concentration was increased from 0 to 50 mg/L, the CBZ removal efficiency by the Co3@NCNT-800/PMS system exhibited a marked decline from 64.7% to 48.0%, 22.3%, and 13.3%. This inhibitory effect of HA is attributed to the competitive adsorption on the catalytic surface [53], as well as the interactions between HA and free radicals, which result in a decreased concentration of radicals available for the reaction with CBZ.

### 2.4. Recycling Studies

**Figure 7:**
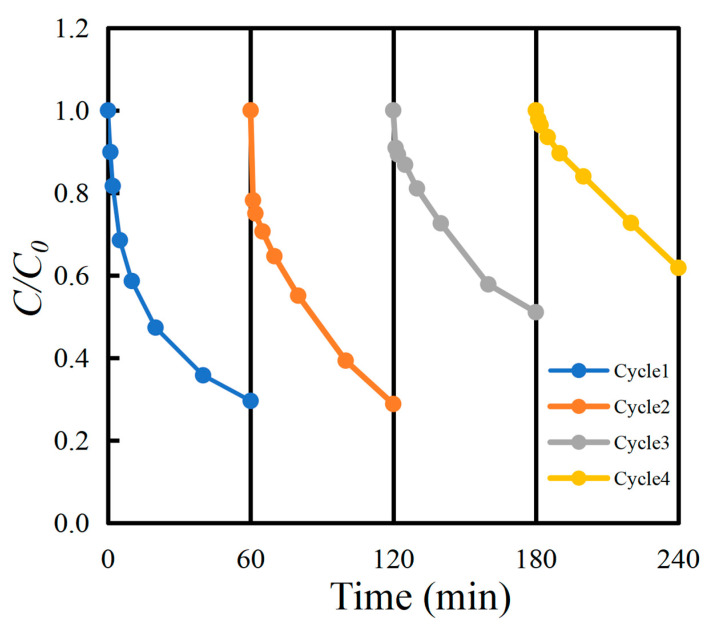
Repeatability of Co3@NCNT-800 for four cycling tests.

The recycling and stability of Co3@NCNT-800 activation with the optimized dosages were evaluated through four cycles of experiments. Following the reaction, the catalyst was washed several times with distilled water and dried. Then, the regenerated catalyst was used to catalyze the activation of PMS for CBZ degradation under identical experimental conditions. The outcomes of this process are depicted in Figure 7. A discernible trend is evident from the figure: The degradation efficiency of CBZ diminishes as the number of catalyst reuse cycles increases. This reduction in efficiency could be ascribed to the partial dissolution of Co_3_O_4_ from the catalyst, coupled with a decrease in cobalt content during the recycling experiments. In addition, not all intermediate products generated during CBZ degradation are completely removed; some of these products may be adsorbed onto the catalytic surface, thereby blocking some of the active sites on Co3@NCNT-800. This blockage could inhibit the reaction between CBZ and PMS, resulting in a low degradation efficiency. However, after four cycles, the Co3@NCNT-800 catalyst can still degrade approximately 38.2% of the CBZ present in the system, which is 59% of the initial degradation efficiency. These findings underscore the robust recyclability, stability, and cost-effectiveness of the Co3@NCNT-800 catalyst, making it a viable option for environmental cleanup efforts.

### 2.5. Main Reactive Oxygen Species (ROS) of the Co3@NCNT-800/PMS System

**Figure 8:**
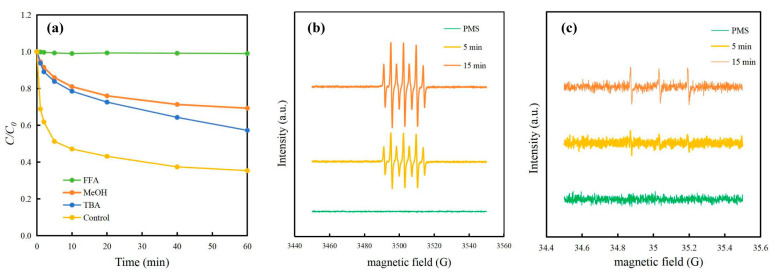
Quenching agents on CZB degradation (**a**); the ESR spectra with the addition of DMPO (**b**) and TEMP (**c**).

**Figure 9:**
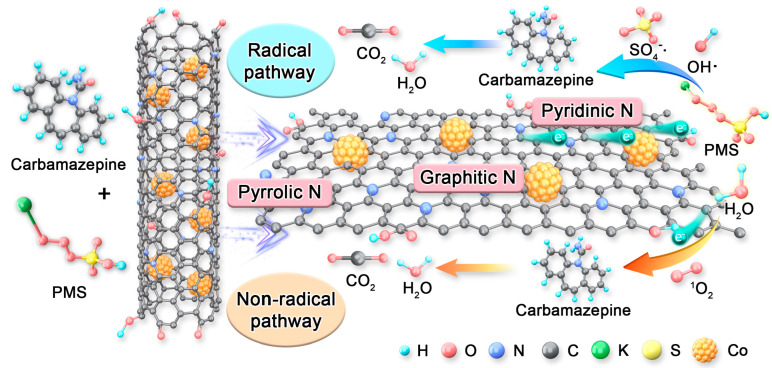
The proposed reaction mechanism for CBZ degradation by Co3@NCNT-800/PMS system.

This study delineated the primary ROS present in the reaction system by using radical scavenging assays and EPR spectroscopy. The results of the scavenging experiments (Figure 8a) reveal that the performance of the Co3@NCNT-800/PMS system is influenced by the addition of methanol (MeOH) or TBA. The attenuation in CBZ degradation upon the introduction of these scavengers indicates that SO_4_•^−^ and hydroxyl radicals (•OH) are active participants in the reaction mechanism [54]. The substantial inhibition of CBZ degradation following the addition of furfuryl alcohol (FFA) points to singlet oxygen (^1^O_2_) as another predominant ROS within the system. This hypothesis was further substantiated by the direct detection of ROS generated in the system through EPR spectroscopy, which provided additional insight into the underlying chemical dynamics. Analysis of the data presented in Figure 8b elucidates that PMS alone does not yield a detectable DMPOX signal. However, upon integration with Co3@NCNT-800, significant DMPOX signals were discernible at 5 and 15 min, indicating the generation of hydroxyl (•OH) and SO_4_•^−^. The EPR spectrum exhibited seven principal peaks associated with the DMPO-X adducts, which is attributed to the rapid oxidation of the highly unstable DMPO-OH or DMPO-SO_4_ adducts by ROS [55,56]. Corroborating these findings with the radical scavenging experiments indicates that although •OH and SO_4_•^−^ are produced, they are not the predominant reactive species in this context. Furthermore, the detection of the characteristic signal peak of TEMP-^1^O_2_ (Figure 8c), where TEMP specifically interacts with singlet oxygen (^1^O_2_), confirms the presence of ^1^O_2_ [57]. Consequently, the collective evidence posits that within the Co3@NCNT-800/PMS system, ^1^O_2_ is the primary ROS responsible for CBZ degradation, while the contribution of other ROS is supplementary. This conclusion is pivotal for understanding the mechanistic pathway of CBZ degradation and the role of ROS in AOPs. The proposed reaction mechanism is illustrated in Figure 9.

### 2.6. Analysis of the Intermediates and Pathways of CBZ Degradation

**Scheme 1:**
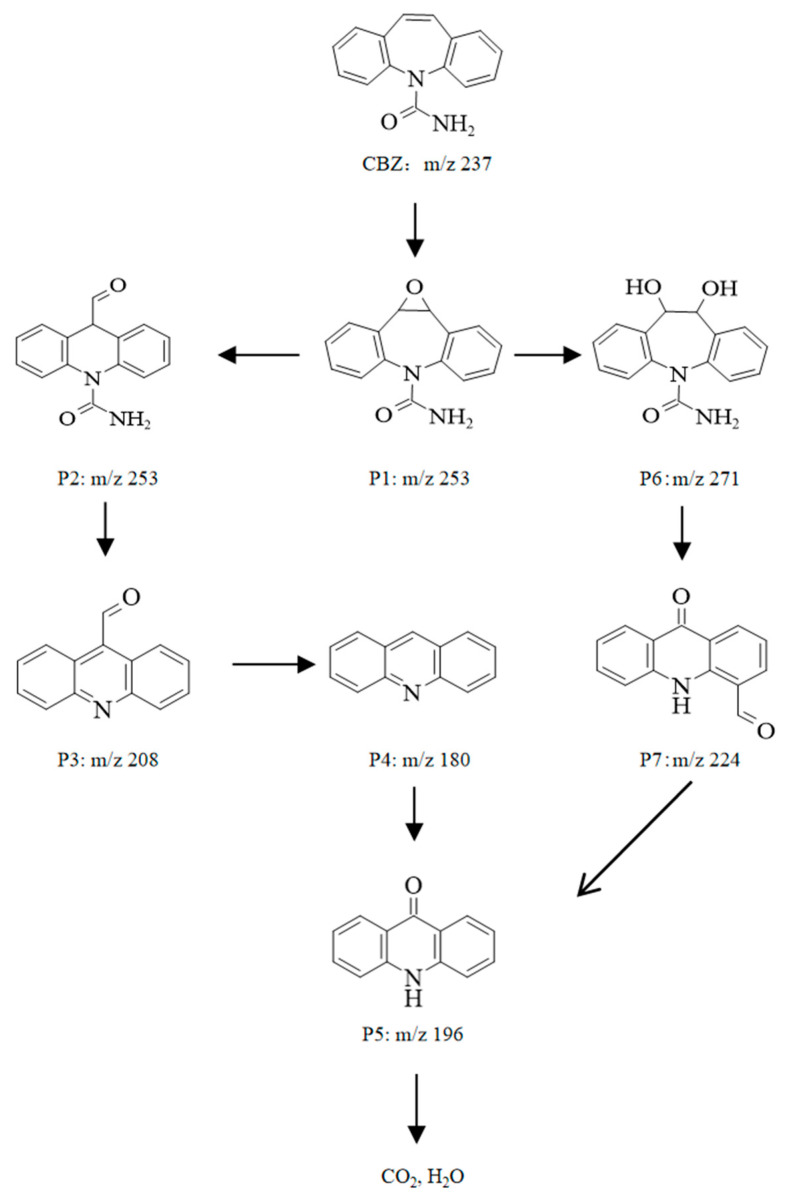
The possible pathway of CBZ degradation in Co3@NCNT-800/PMS system.

Using LC–MS, the CBZ degradation intermediates of the Co3@NCNT-800/PMS system were identified. The corresponding mass spectra are shown in Figure S3 and Scheme 1, which depict the proposed degradation pathways. Two distinct degradation routes were hypothesized for CBZ. In Pathway I, the initial step involves the attack of the 3C site on intermediate P1 by ROS, leading to a ring contraction reaction that yields P2 with a mass-to-charge ratio (*m*/*z*) of 253. Subsequently, P2 undergoes acetylation to form P3 (*m*/*z* = 208) in the presence of singlet oxygen (^1^O_2_) and SO_4_•^−^. Then, the aldehyde group of P3 is cleaved, resulting in the formation of P4 (*m*/*z* = 180), which is further oxidized to produce P5 (*m*/*z* = 196). Pathway II starts with the hydrolysis of the epoxide bond in P1, generating P6 (*m*/*z* = 271), followed by the cleavage and oxidation of the C–C bond to yield P7 (*m*/*z* = 224). P7 undergoes further deoxygenation to form P5. The final stage in both pathways involves the continued attack on P5 by ROS, culminating in the mineralization of the molecules into small molecules and its eventual transformation into CO_2_ and H_2_O.

## 3. Materials and Methods

### 3.1. Preparation of Co–N Co-Doped CNTs

Bean dregs were procured from a local tofu vendor in Cixi City, China. The bean dregs were initially desiccated overnight at 110 °C. Subsequently, they were subjected to pyrolysis in a muffle furnace at 400 °C, with a ramping rate of 10 °C/min, for 2 h. The resultant carbonized material is hereafter referred to as BDC. Considering that zinc chloride (ZnCl_2_) is known to catalyze the formation of porous carbon structures, a stoichiometric mixture of BDC and ZnCl_2_ was prepared at a 1:1 mass ratio. This mixture was then thermally treated in a tubular furnace under a nitrogen atmosphere at 500 °C for 1 h. The resulting sample was washed thoroughly with 0.1 M hydrochloric acid (HCl) solution and deionized water until the filtrate reached a neutral pH. After washing, the sample was dried overnight at 110 °C. The final product, denoted as BZ, represents the AC derived from this process.

The Co@NCNT catalysts were synthesized using cobalt chloride as the catalytic precursor. Initially, 5 g of dicyandiamide was dissolved in 100 mL of CoCl_2_ aqueous solution under continuous stirring. Next, 0.5 g of the previously synthesized BZ was introduced into the cobalt solution, and the mixture was stirred for 1 h. The resultant mixture was then dried at 90 °C for 12 h. The dried powder underwent precarbonization in a muffle furnace at 400 °C, with a heating rate of 5 °C/min, for 2 h. Subsequent annealing was performed in a tubular furnace at various temperatures (700 °C, 800 °C, and 900 °C) for 1 h under a nitrogen atmosphere. The annealed powders were then washed with 1 M HCl solution and deionized water until a neutral pH of the filtrate was achieved, followed by overnight drying at 110 °C. The final catalysts were designated as CoX@NCNT-y, where “y” represents the annealing temperatures of 700 °C, 800 °C, and 900 °C, and “x” corresponds to the initial molar concentrations of CoCl_2_ (0 mM, 1 mM, and 3 mM) used in the synthesis, yielding Co0@NCNT-800, Co1@NCNT-800, and Co3@NCNT-800, respectively.

### 3.2. Characterization

The morphological characteristics of the CNTs were examined using SEM (ZEISS Sigma 300, Oberkochen, Germany) and TEM (JOEL JEM-2100F, Akishima, Japan). The elemental composition of C, H, N, and O in the CNTs was determined using an Elementar UNICUBE analyzer (UNICUBE, Elementar, Langenselbold, Germany). The textural properties, including the mesoporous volume (V_meso_), BET specific surface area (S_BET_), and microporous volume (V_micro_), were quantified at −196 °C using an ASAP 2020 specific surface area and porosity analyzer (Micromeritics, Norcross, GA, USA) via N_2_ adsorption/desorption isotherms. The chemical composition and electronic states of the surface elements of the CNTs were probed using XPS (Thermo Scientific K-Alpha, Waltham, MA, USA). The crystalline structure of the CNTs was elucidated by XRD, which was conducted on a Rigaku Ultima IV diffractometer. For the quantitative analysis of CBZ, high-performance liquid chromatography (HPLC, Agilent 1260 Infinity II, Santa Clara, CA, USA) was used. Furthermore, the degradation products of CBZ were characterized by coupling HPLC with mass spectrometry (HPLC-MS, Agilent 1290 UPLC-QTOF 6550, USA).

### 3.3. Calculation Methods

Batch experiments were systematically performed to evaluate the catalytic performance of CNTs in the activation of PMS for the degradation of CBZ. A 100 mL aliquot of CBZ solution at a concentration of 20 mg/L was transferred into a conical flask, followed by the sequential addition of PMS and 0.02 g of catalyst. Then, the mixture was stirred at 25 °C with a rate of 200 rpm. When necessary, the solution pH was adjusted to the desired value using 0.1 mmol/L H_2_SO_4_ and 0.1 mmol/L NaOH solutions. At the predetermined time intervals, 1.0 mL of the reaction mixture was withdrawn using a syringe. The sample was then filtered through a 0.22 µm aqueous microporous filter into a vial containing 2 µL of anhydrous methanol. After vigorous shaking to ensure mixing, the sample was subjected to HPLC. The degradation efficiency of CBZ is calculated using Equation (1).

(1) $$\mathrm{Degradation}\;\mathrm{rate}\;\mathrm{of}\;\mathrm{CBZ}=C/C_{0},$$

where C_0_ and C are the initial and residual concentrations of CBZ, respectively.

In elucidating the effects of various parameters on the degradation of CBZ, controlled static batch experiments were meticulously designed, with varying conditions such as temperature, Co-doping ratio, catalyst and PMS dosages, reaction pH, HA concentration, and the presence of coexisting anions. Furthermore, radical scavenging experiments were performed to discern the types of reactive species generated by CNTs when PMS is activated during CBZ degradation. Before degradation, specific amounts of scavengers were introduced into the solution to qualitatively identify the predominant reactive species formed by the PMS-activated catalyst, as evidenced by their suppression effect on the degradation of CBZ. Hydroxyl radicals (•OH) were quenched using tert-Butanol; SO_4_•^−^ were quenched using methanol (MeOH), and singlet oxygen (^1^O_2_) was quenched using furfuryl alcohol (FFA). In addition, EPR spectroscopy was conducted to further verify the reactive species produced upon activation, and the capture agents were DMPO and TEMP. In the CNT reuse experiment, Co3@NCNT-800 was isolated from the suspension using a 0.45-μm pore-sized filter membrane. The material was then subjected to multiple rinses with ultrapure water to ensure thorough removal of residual contaminants. After washing, the catalyst was dried at 110 °C for 12 h. This procedure was used to prepare Co3@NCNT-800 for reuse in subsequent experimental cycles.

## 4. Conclusions

In this investigation, cobalt–nitrogen-doped carbon nanotubes (Co3@NCNT-800) were synthesized using a straightforward and cost-effective approach. The application potential of these nanotubes for the degradation of water pollutants was assessed through a series of characterization analyses and the activation of PMS for CBZ degradation. The findings of this study indicate that cobalt serves as a catalyst for the formation of CNTs and enhances their graphitization. The presence of cobalt and nitrogen significantly augments the catalytic degradation efficiency of Co3@NCNT-800, achieving a CBZ degradation rate of 64.7% within 60 min. The catalyst exhibits broad pH adaptability, robust stability, and reusability. The Co3@NCNT-800 demonstrates superior efficiency in facilitating the degradation of carbamazepine with PMS compared to conventional catalysts. However, the presence of HA in the solution inhibits the degradation process. Mechanistic studies revealed that the oxidative degradation of CBZ proceeded via the free radical and nonradical pathways, with singlet oxygen ^1^O_2_ playing a predominant role. The potential degradation pathways of CBZ were further elucidated using LC–MS. Consequently, the synthesized Co3@NCNT-800 catalyst shows great application potential in the degradation of water pollutants.

## Figures and Tables

**Table 1:** Textural and surface properties and elemental composition of each prepared sample.

Sample	C	H	N	O	SBET	Vtotal	Vmicro	Vmeso
	(%)	(%)	(%)	(%)	(m^2^·g^−1^)	(cm^3^·g^−1^)	(cm^3^·g^−1^)	(cm^3^·g^−1^)
Co0@NCNT-800	70.5	1.2	10.8	11.3	677.2	0.386	0.287	0.099
Co1@NCNT-800	70.1	1.2	11.5	10.4	715.4	0.405	0.295	0.110
Co3@NCNT-800	67.5	1.1	11.5	9.6	614.0	0.322	0.252	0.070

**Table 2:** Summary of PMS activation by catalysts to remove CBZ.

Catalysts (g/L)	Reaction Conditions	CBZ (mg/L)	Removal Efficiency	Ref.
A-SiO_2_/TiO_2_ (1 g/L)	[PMS] = 0.02 mmol/L, T = 30 °C	20.0 mg/L	>35% (60 min)	[46]
g-C_3_N_4_ (1 g/L)	[PMS] = 5 mmol/L, T = room temperature	5.0 mg/L	>15% (60 min)	[47]
g-C_3_N_4_ doping with ammonium oxalate (OCN) (1 g/L)	[PMS] = 5 mmol/L, T = room temperature	5.0 mg/L	>36% (60 min)	[47]
TiO_2_-400 (0.10 g/L)	[PMS] = 1 mmol/L, T = 20 °C	10.0 mg/L	>33% (60 min)	[48]
Bi_2_WO_6_ under the irradiation of xenon lamp (1 g/L)	[PMS] = 0.5 g/L, T = 25 °C	10.0 mg/L	>60% (60 min)	[49]
α-SnWO4/ZnO nanomaterial (1 g/L)	[PMS] = 0.6 g/L	10.0 mg/L	>20% (60 min)	[50]
α-MnO_2_ (2 mmol/L)	[PMS] = 0.1 g/L	5 mg/L	>64% (30 min)	[51]
Co3@NCNT-800 (0.2 g/L)	[PMS] = 1.0 mmol/L, T = room temperature	20.0 mg/L	64.7% (60 min)	This work

## Data Availability

Data are contained within the article and Supplementary Materials.

## Supplementary Materials

The following supporting information can be downloaded at https://www.mdpi.com/article/10.3390/molecules29071525/s1, Figure S1: XPS spectra of Co3@NCNT-800 (before reaction) and Co3@NCNT-800 (after reaction); Figure S2: Nitrogen adsorption isotherm of Co0@NCNT-800 (a), Co1@NCNT-800 (b), and Co3@NCNT-800 (c); Figure S3: Extraction of mass spectra with different retention time (tr); Table S1: Comparative analysis of the surface elemental composition and content of Co3@NCNT-800 before and after the catalytic reaction; Table S2: The quantitative changes in nitrogen and cobalt configurations of Co3@NCNT-800 before and after the catalytic reaction.
