# Supplementary material for: Preparation of Cobalt–Nitrogen Co-Doped Carbon Nanotubes for Activated Peroxymonosulfate Degradation of Carbamazepine
Source: Molecules. 2024 Mar 28;29(7):1525. doi: 10.3390/molecules29071525 (PMC11013098; doi:10.3390/molecules29071525)
Supplement: Supplementary file 1 [file molecules-29-01525-s001.zip › molecules-2914458-supplementary.pdf]

Supporting Information:

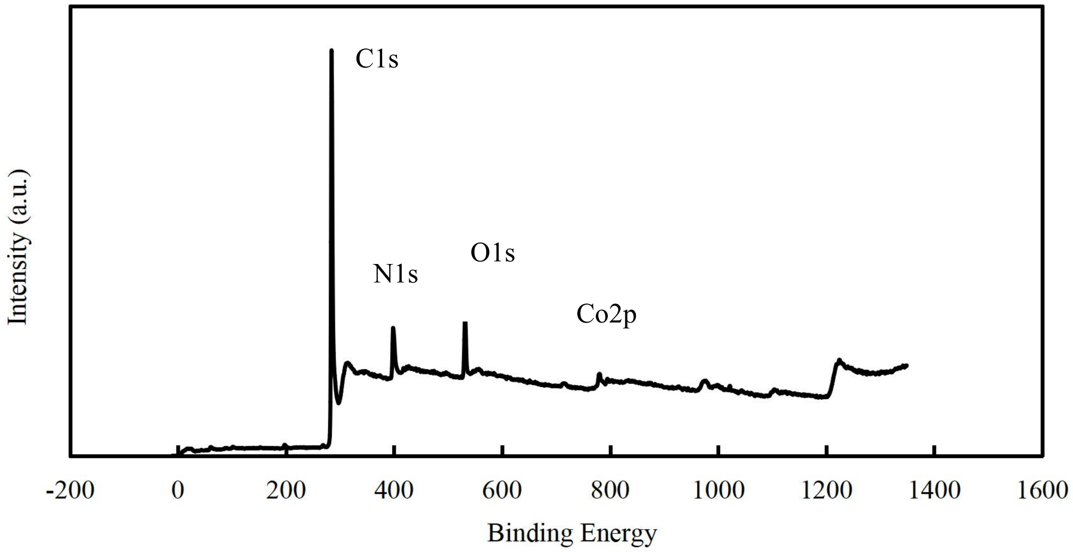

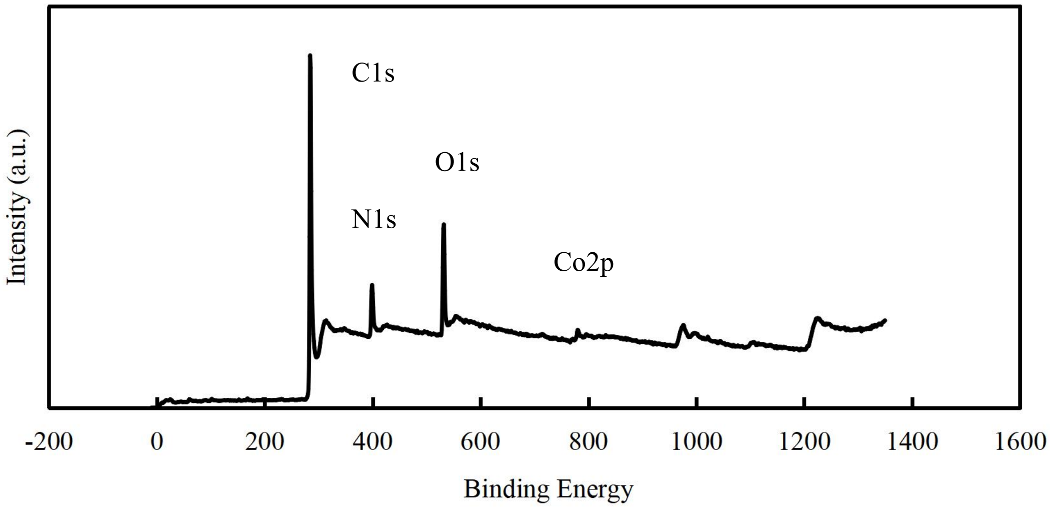

Figure S1. XPS spectra of Co3@NCNT-800 (before reaction) and Co3@NCNT-800 (after reaction).

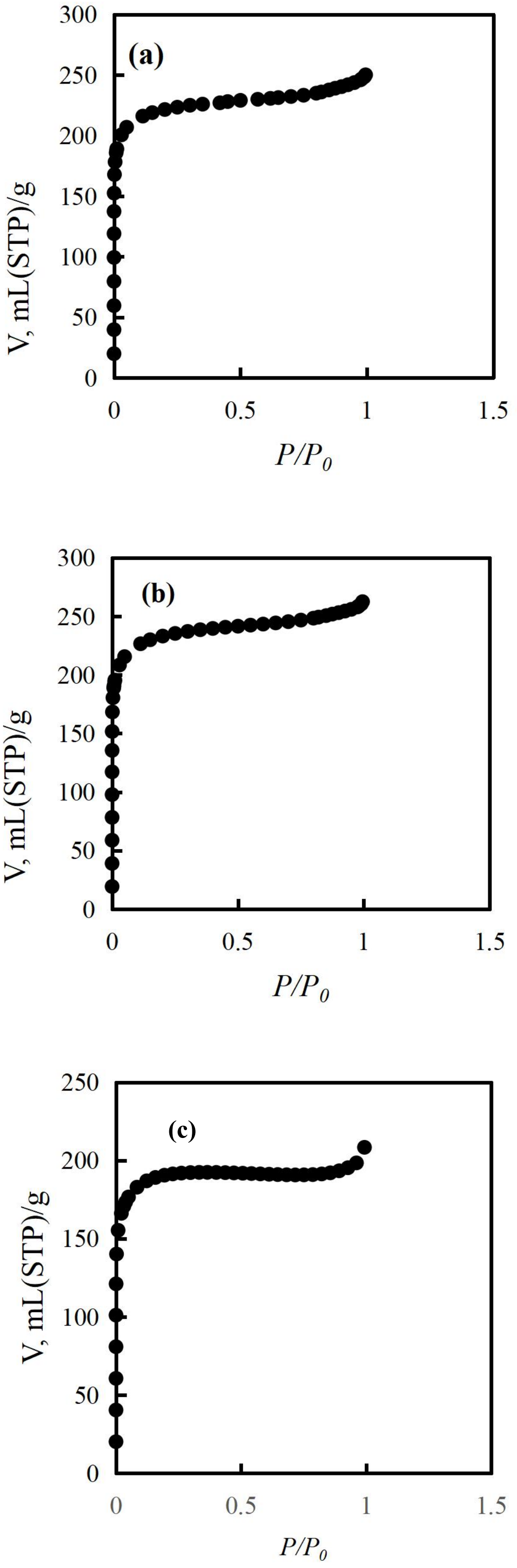

Figure S2. Nitrogen adsorption isotherm of Co0@NCNT-800 (a), Co1@NCNT-800 (b), and Co3@NCNT-800 (c).

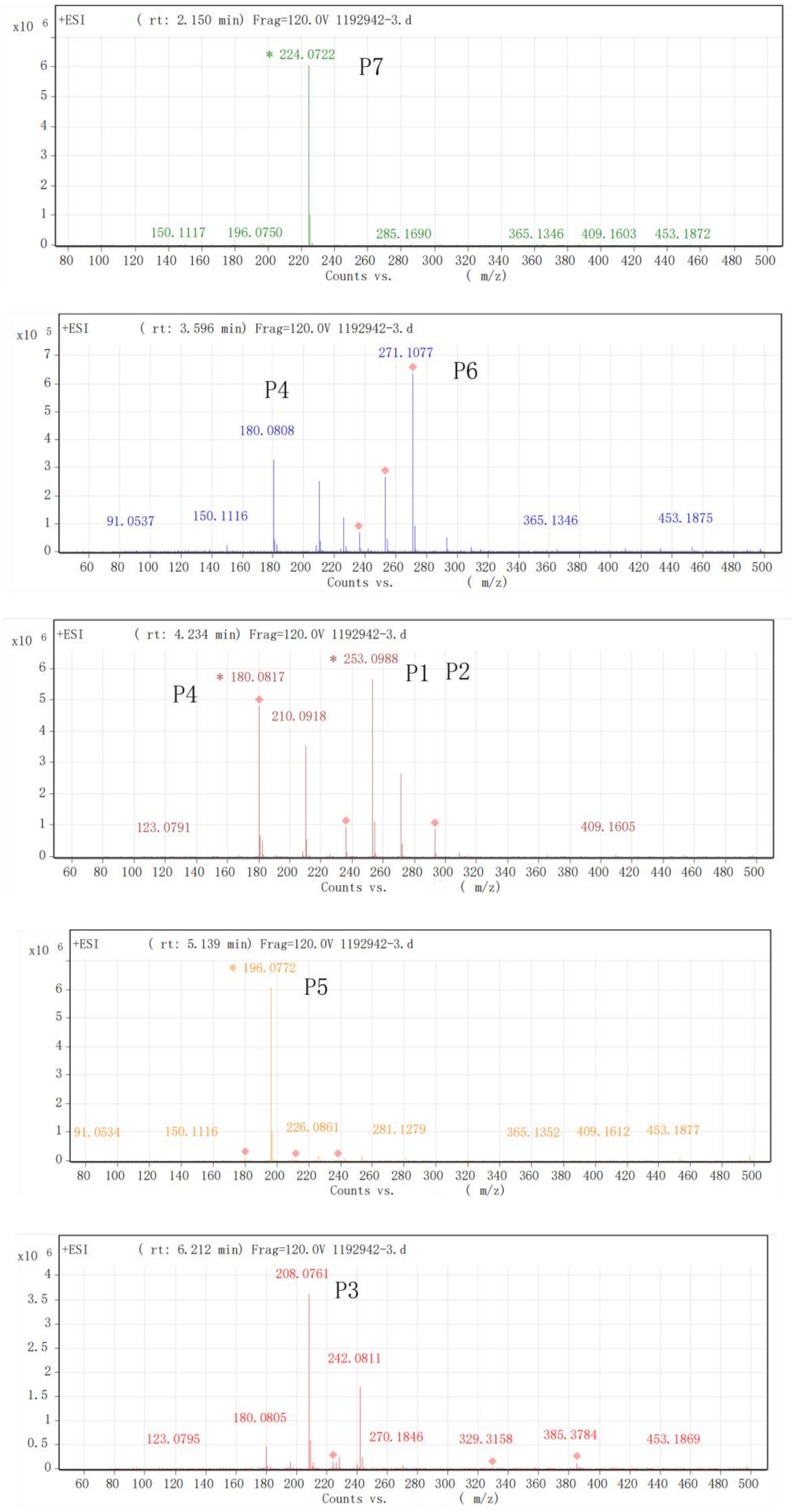

Figure S3. Extraction of mass spectra with different retention time (tr).

Table S1. Comparative analysis of the surface elemental composition and content of Co3@NCNT-800 before and after the catalytic reaction

	C1s (%)	N1s (%)	O1s (%)	Co2p (%)
Co3@NCNT-800 (before reaction)	83.8	9.4	5.8	0.6
Co3@NCNT-800 (after reaction)	79.4	8.7	11.2	0.5

Table S2. The quantitative changes in nitrogen and cobalt configurations

of Co3@NCNT-800 before and after the catalytic reaction

	pyridine N (%)	pyrrolic N (%)	graphitic N (%)	CoO (%)	Co_3O_4 (%)
Co3@NCNT-800 (before reaction)	58.26	34.72	7.03	86.17	13.83
Co3@NCNT-800 (after reaction)	55.88	37.44	6.67	95.76	4.24
